# Oseltamivir- and Amantadine-Resistant Influenza Viruses A (H1N1)

**DOI:** 10.3201/eid1506.081357

**Published:** 2009-06

**Authors:** Peter K.C. Cheng, Tommy W.C. Leung, Eric C.M. Ho, Peter C.K. Leung, Anita Y.Y. Ng, Mary Y.Y. Lai, Wilina W.L. Lim

**Affiliations:** Centre for Health Protection, Hong Kong Special Administrative Region, People’s Republic of China

**Keywords:** Influenza, antimicrobial resistance, viruses, H1N1, respiratory infections, zoonoses, genetic reassortment, spontaneous mutation, Hong Kong, dispatch

## Abstract

Surveillance of amantadine and oseltamivir resistance among influenza viruses was begun in Hong Kong in 2006. In 2008, while both A/Brisbane/59/2007-like and A/Hong Kong/2652/2006-like viruses (H1N1) were cocirculating, we detected amantadine and oseltamivir resistance among A/Hong Kong/2652/2006-like viruses (H1N1), caused by genetic reassortment or spontaneous mutation.

A high rate of oseltamivir resistance in seasonal influenza virus A (H1N1) infection was reported in Europe in the winter of 2007–08 (*1*). Of subtype H1N1 isolates tested in 18 European countries, 59 (13.5%) of 437 were resistant to oseltamivir and carried the substitution of histidine by tyrosine at residue 274 (H274Y) of the neuraminidase (NA) gene. Genetic analysis showed that all oseltamivir-resistant strains of subtype H1N1 virus remained susceptible to amantadine.

In Hong Kong Special Administrative Region, People’s Republic of China, amantadine and oseltamivir are not commonly used to treat patients with influenza. Surveillance of amantadine and oseltamivir resistance among influenza viruses was begun in 2006 after a high rate of amantadine resistance was reported among subtype H3N2 viruses (*2*) and a stockpile of oseltamivir was purchased for pandemic preparedness. Oseltamivir resistance was first detected in January 2008. We report detection of subtype H1N1 virus isolates that became resistant to amantadine and oseltamivir because of genetic reassortment and spontaneous mutation.

## The Study

As part of ongoing surveillance, respiratory samples are routinely collected from patients seeking treatment with respiratory illness in clinics and hospitals of Hong Kong. Virus isolation is performed according to a standard protocol (*3*). From January 2006 through June 2008, a total of 827 influenza viruses (H1N1) were tested for oseltamivir resistance by an enzyme inhibition assay (NA-STAR; Applied Biosystems, Foster City, CA, USA) or by nucleotide sequencing of the NA gene to detect the H274Y mutation. The isolates were also tested for resistance to amantadine by an in-house–designed PCR (protocol available on request) and nucleotide sequencing of the matrix (M2) gene segment. A 575-nt fragment of the NA gene and a 199-nt fragment of the M2 gene were amplified by the designed primers N1–1H and N1–2H, and MA 692 and MA 891 (available on request), respectively, and sequenced by using a Genetic Sequencer 3100 or 3130XL (Applied Biosystems). Sequences obtained were aligned by using Simmonics (*4*) and analyzed by MEGA version 2.1 (*5*). Increasing amantadine resistance, from 107 (48.0%) of 223 isolates in 2006 to 12 (85.7%) of 14 in 2007, was associated with clade 2C A/Hong Kong/2652/2006-like viruses (*6*); none were resistant to oseltamivir.

A subtype H1N1 virus resistant to oseltamivir, but sensitive to amantadine, was first detected in Hong Kong in January 2008, which coincided with the emergence of clade 2B A/Brisbane/59/2007-like viruses. From January through June 2008, 87 (12.5%) of 697 isolates tested were resistant to oseltamivir. They all carried the H274Y mutation in the NA gene and showed an ≈1,000-fold reduction in susceptibility to oseltamivir (50% inhibitory concentration values increased from 0.5 nmol/L to 500 nmol/L). Analysis of the M2 genes showed that 3 (3.4%) of 87 oseltamivir-resistant isolates also carried the S31N mutation associated with amantadine resistance. These 3 viruses were isolated from 2 infants and an elderly woman in different hospitals during 2008 (March, April, and June, respectively). To eliminate the possibility of mixed strains, we designed a real-time PCR using single nucleotide polymorphisms to detect antimicrobial drug–susceptible strains (with S31 and H274) and drug resistant strains (with S31N and H274Y) in the samples (method available on request). Results showed that the 3 isolates did not possess S31 and H274.

To further study the genetic mechanism of emergence of antiviral drug resistance among subtype H1N1 viruses, we performed nucleotide sequencing on a partial segment of the hemagglutinin (HA) gene on 84 (97%) of 87 oseltamivir-resistant and 37 (5%) of 697 oseltamivir-susceptible viruses isolated in our laboratory in 2008. PCR and DNA sequencing of the HA gene were carried out by using the primers H1–1 and H1–2, which flank a fragment of 612 nt of the HA segment (*7*). Sequences were obtained from 121 virus isolates (Table).

**Table:** Phylogenetic analysis of HA, NA, and M2 genes of influenza viruses A (H1N1) with different drug-resistance characteristics isolated in Hong Kong, January–June 2008*

Characteristics	No. isolates	HA clade	NA clade	M2 clade	Mutation
Oseltamivir resistant (n = 84)					
Amantadine susceptible	81	2B†	2B	2B	H274Y in NA
Amantadine resistant	1	2C†	2B	2C	H274Y in NA, S31N in M2
	2	2C	2C	2C	H274Y in NA, S31N in M2
Oseltamivir susceptible (n = 37)					
Amantadine susceptible	20	2B	2B	2B	None
Amantadine resistant	14	2C	2C	2C	S31N in M2
	3	2B	2B	2C	S31N in M2

*HA, hemagglutinin; NA, neuraminidase; M2, matrix. †Clade 2B or clade 2C indicates the gene specified has a nucleotide homology >99% to that of A/Brisbane/59/2007 or A/Hong Kong/2652/2006 virus, respectively.

Of 37 oseltamivir-susceptible viruses, analysis of the HA, NA, and M2 genes showed that 20 were susceptible to amantadine and similar to the clade 2B A/Brisbane/59/2007 virus (GenBank accession no. CY030232) (*8*). Fourteen were similar to clade 2C A/Hong Kong/2652/2006 virus (GenBank accession no. CY031342), an amantadine-resistant virus isolated in our laboratory in 2006 which remained prevalent in Hong Kong in 2007 and 2008. Three isolates had HA and NA genes similar to those of clade 2B but carried an amantadine-resistant M2 gene similar to that of the A/Hong Kong/2652/2006 virus.

Among 84 oseltamivir-resistant strains, 81 remained susceptible to amantadine and had HA and M2 genes similar to those of clade 2B A/Brisbane/59/2007 virus but carried a single point mutation H274Y in the NA gene. The other 3 isolates were resistant to oseltamivir and amantadine; they had similar HA and M2 genes to those of clade 2C A/Hong Kong/2652/2006 virus but had the H274Y mutation in their NA gene. One of the viruses had an NA gene similar to that of the oseltamivir-resistant clade 2B A/Brisbane/59/2007 virus, whereas the other 2 viruses had NA genes similar to those of amantadine-resistant clade 2C A/Hong Kong/2652/2006 virus (Figure). These results suggest that the 3 isolates acquired amantadine and oseltamivir resistance by different mechanisms. A clade 2C A/Hong Kong /942/2008 virus acquired an NA gene carrying the H274Y mutation by reassortment with an oseltamivir-resistant clade 2B virus; the other 2 clade 2C viruses, A/Hong Kong /1052/2008 and A/Hong Kong /1313/2008, acquired oseltamivir resistance through a spontaneous H274Y mutation in the NA gene.

**Figure:**
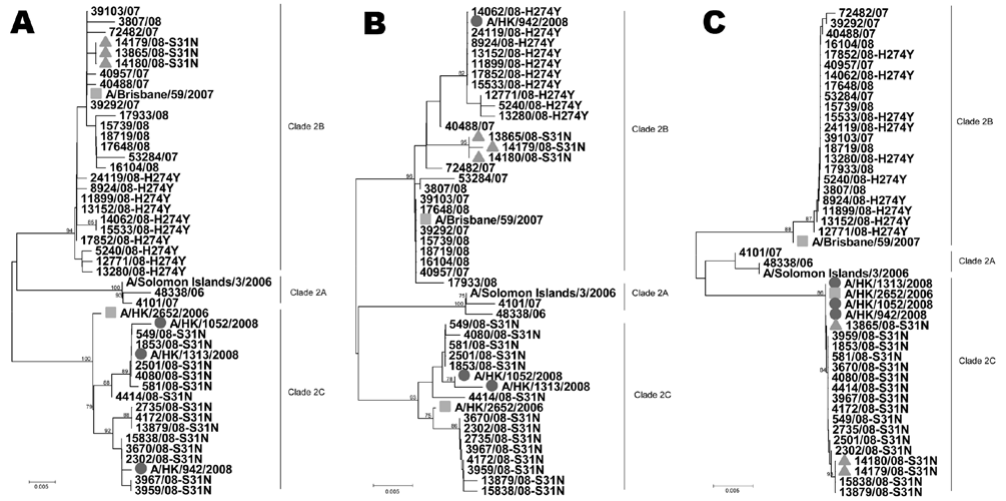
Phylogenetic analysis of the hemagglutinin gene (A), neuraminidase gene (B), and matrix gene (C) of influenza virus (H1N1). The 3 clade 2C viruses with double resistance are indicated by circles, the 3 clade 2B viruses with amantadine resistance are indicated by triangles, and A/Brisbane/59/2007 and A/Hong Kong/2652/2006 are indicated by squares. Scale bars indicate 0.5% difference in nucleotide sequence.

To study the effect of the resistant gene on the replication efficiency of the viruses, we conducted experiments using amantadine- and oseltamivir-resistant viruses in MDCK cells. Briefly, we placed in quadruplicate on MDCK cells one 50% tissue culture infectious dose per 0.1 mL of subtype H1N1 viruses with different antiviral drug–resistance patterns (amantadine-resistant only, oseltamivir-resistant only, amantadine- and oseltamivir-resistant, and amantadine- and oseltamivir-susceptible), incubated the cultures at 33°C, and examined the cytopathic effect (CPE) daily for 7 days. By observing the timing of CPE appearance and relative progressive rate of CPE, we found no substantial difference in growth efficiency and similar replication patterns for the 4 different types of subtype H1N1 strains.

## Conclusions

Reassortment of gene segments likely occurred among clade 2B and clade 2C subtype H1N1 viruses while they were cocirculating in Hong Kong. Clade 2B A/Brisbane/59/2007-like viruses also were cocirculating with clade 2C A/Hong Kong /2652/2006-like viruses in about the same proportion during the 2008 influenza season. We identified 3 clade 2B A/Brisbane/59/2007-like viruses (isolated from 1 outbreak) that were oseltamivir-sensitive in their HA and NA genes but carried an amantadine-resistant M2 gene that was similar to clade 2C A/Hong Kong/2652/2006 virus. We also identified 3 virus isolates that were resistant to both amantadine and oseltamivir. The HA, NA, and M2 gene sequences of the 3 oseltamivir- and amantadine-resistant subtype H1N1 isolates were deposited in GenBank (accession nos. FJ227684–FJ227689 and FJ581446–FJ581448).

All 3 isolates were clade 2C A/Hong Kong/2652/2006-like viruses, but they acquired oseltamivir resistance through spontaneous mutation or reassortment with a clade 2B A/Brisbane/59/2007-like virus. Gene sequencing results confirmed that no epidemiologic link existed among the 3 patients infected with these isolates.

Monitoring of antiviral resistance among influenza isolates showed that resistance pattern changed among subtype H1N1 viruses when different lineages were introduced. While oseltamivir resistance appears to be largely confined to clade 2B viruses and amantadine resistance to clade 2C viruses, oseltamivir- and amantadine-resistant viruses due to spontaneous mutation or reassortment were detectable when clade 2B and 2C viruses were cocirculating. If antiviral resistance markers are combined with sequence data of HA, NA, and M genes, systematic monitoring would make it possible to track the spread of influenza viruses globally and to clarify the underlying mechanism for the spread of such resistance.

## References

[1] Lackenby A, Hungnes O, Dudman SG, Meijer A, Paget WJ, Hay AJ, Emergence of resistance to oseltamivir among influenza A(H1N1) viruses in Europe. Euro Surveill. 2008;13:1–2. 10.2807/ese.13.05.08026-en 18445375

[2] Bright RA, Medina MJ, Xu X, Perez-Oronoz G, Wallis TR, Davis XM, Incidence of adamantane resistance among influenza A (H3N2) viruses isolated worldwide from 1994 to 2005: a cause for concern. Lancet. 2005;366:1175–81. 10.1016/S0140-6736(05)67338-2 16198766

[3] Lennette EH, Lennette DA, Lennette ET, eds. Diagnostic procedures for viral, rickettsial and chlamydial infections, 7th ed. Washington: American Public Health Association; 1995.

[4] Simmonds P, Smith DB. Structural constraints on RNA virus evolution. J Virol. 1999;73:5787–94. 10364330 10.1128/jvi.73.7.5787-5794.1999 PMC112639

[5] Kumar S, Tamura K, Jakobsen IB, Nei M. MEGA2: Molecular Evolutionary Genetics Analysis software. Tempe (AZ): Arizona State University; 2001. 10.1093/bioinformatics/17.12.1244 11751241

[6] World Influenza Centre, Medical Research Council. Influenza between October 2007 to July 2008 [cited 2009 Jan 12]. Available from http://www.nimr.mrc.ac.uk/wic

[7] Wright KE, Wilson GA, Novosad D, Dimock C, Tan D, Weber JM. Typing and subtyping of influenza viruses in clinical samples by PCR. J Clin Microbiol. 1995;33:1180–4. 7615726 10.1128/jcm.33.5.1180-1184.1995 PMC228127

[8] World Health Organization. Recommended composition of influenza virus vaccines for use in the 2008–2009 northern hemisphere influenza season [cited 2008 Oct 9]. Available from http://www.who.int/csr/disease/influenza/recommendations2008_9north/en/index.html

